# Theoretical and Numerical Study on Stress Intensity Factors for FRP-Strengthened Steel Plates with Double-Edged Cracks

**DOI:** 10.3390/s18072356

**Published:** 2018-07-20

**Authors:** Hai-Tao Wang, Gang Wu, Yu-Yang Pang

**Affiliations:** 1 College of Civil and Transportation Engineering, Hohai University, Nanjing 210098, China; cewht@hhu.edu.cn; 2 Key Laboratory of Concrete and Prestressed Concrete Structures of the Ministry of Education, Southeast University, Nanjing 210096, China; 230149376@seu.edu.cn

**Keywords:** stress intensity factor, double-edged cracks, steel plate, fiber reinforced polymer (FRP), strengthening, finite element simulation

## Abstract

This paper presents a theoretical and numerical study on the stress intensity factors for double-edged cracked steel plates strengthened with fiber reinforced polymer (FRP) plates. Based on the stress intensity factor solution for infinite center-cracked steel plates strengthened with FRP plates, expressions of the stress intensity factors were proposed for double-edged cracked steel plates strengthened with FRP plates by introducing two correction factors: *β* and *f*. A finite element (FE) simulation was carried out to calculate the stress intensity factors of the steel plate specimens. Numerous combinations of the specimen width, crack length, FRP thickness and Young’s modulus, adhesive thickness, and shear modulus were considered to conduct the parametric investigation. The FE results were used to investigate the main influencing factors of the stress intensity factors and the correction factor, *β*. The expression of the correction factor, *β*, was formulated and calibrated based on the FE results. The proposed expressions of the stress intensity factors were a function of the applied stress, the crack length, the ratio between the crack length and the width of the steel plate, the stiffness ratio between the FRP plate and steel plate, the adhesive thickness, and the shear modulus. Finally, the theoretical results and numerical results were compared to validate the proposed expressions.

## 1. Introduction

Steel structures subjected to cyclic loading are vulnerable to fatigue damage. Once fatigue cracks initiate, they may propagate at an increasing growth rate and finally cause catastrophic failure of the structures. In order to extend the service life and avoid the fatigue failure of fatigue-damaged steel members, rehabilitation, which can reduce time and economic cost, is preferred to the replacement or reconstruction of the damaged structures [1,2]. As the externally-bonded fiber reinforced polymer (FRP) technique has been widely used to repair or strengthening concrete structures and masonry structures in the past several decades, this technique has proven to be a promising alternative to the traditional strengthening technique [3,4,5,6,7,8,9]. In recent years, this technique has received much attention in regard to repairing or strengthening fatigue-damaged steel structures [10,11,12]. Many experimental studies have indicated that externally-bonded carbon-FRP (CFRP) laminates can significantly decrease the crack growth rate and extend the fatigue life of cracked steel members [13,14,15,16,17,18,19,20,21,22,23].

For a cracked steel member, the crack growth life is the main contributor to the fatigue life. Based on linear elastic fracture mechanics, the most important parameter in determining the crack growth life is the stress intensity factor which characterizes the magnitude of the singular stress field near the crack tip. Once the value of the stress intensity factor is known, the crack growth rate and crack growth life can be determined based on the crack growth laws of linear elastic fracture mechanics [14,16,18,24,25,26,27,28]. Therefore, calculations on the stress intensity factor of FRP-strengthened cracked steel members are essential for evaluating the fatigue strengthening of cracked steel members.

For some steel members with simple crack configurations and loading, the expressions of the stress intensity factor have been provided in handbooks [29,30]. However, the expressions in the handbooks will no longer be applicable to FRP-strengthened cracked steel members as the strengthening effects of FRP laminates are not considered in the expressions. Currently, numerical methods are usually used to calculate the values of the stress intensity factor for FRP-strengthened cracked steel members. Many finite element (FE) models have been developed, such as the plate-spring model [31], the three-layer plate model [32], the two-layer plate-brick model [33], the 3D brick model [24], the 3D brick-spring-shell model [34], and the 3D brick-spring model [27]. In order to predict the crack growth behavior and fatigue life of an FRP-strengthened cracked steel member, many FE models corresponding to different crack lengths usually need to be developed to calculate the stress intensity factors at different crack lengths [14,16,24,26,27,28]. This procedure is sometimes time-consuming and inconvenient. If theoretical expressions of the stress intensity factors were derived, the crack growth behavior could be evaluated conveniently. This motivated the current authors to develop theoretical expressions of the stress intensity factors for FRP-strengthened cracked steel members.

Nonetheless, investigations on the theoretical expressions for FRP-strengthened cracked steel members have been rather limited. Existing studies have mainly focused on FRP-strengthened center-cracked and single-edged cracked steel plates [34,35,36,37,38]. For an FRP-strengthened cracked steel plate, the stress field at the crack tip becomes more complicated when the effects of the FRP laminates and adhesive layers are considered. An exact analytical solution is an almost intractable task. In order to simplify the analysis, correction factors are usually introduced in the stress intensity factor expressions of unstrengthened steel plates. Several studies have proposed simplified expressions of the stress intensity factor for the center-cracked and single-edged cracked steel plates strengthened with FRP plates [34,35,36,37,38]. According to these equations, the crack growth behavior of FRP-strengthened center-cracked and single-edged cracked steel plates can be predicted expediently. However, to the best knowledge of the investigators, calculation equations of the double-edged cracked steel plates strengthened with FRP plates have not yet been reported in the literature. 

In light of this research gap, this paper investigated the stress intensity factors of double-edged cracked steel plates strengthened with FRP plates with theoretical and numerical methods. The stress intensity factor solution for the infinite center-cracked steel plate strengthened with FRP plates was extended to develop expressions of the stress intensity factors by introducing two correction factors. Then, three-dimensional (3D) FE models were developed to carry out parametrical investigations on the stress intensity factors and the correction factor, *β*. Correction factor *β* was formulated and calibrated based on the FE results. Finally, the proposed expressions were validated by comparing the theoretical and numerical results.

This research shows that the stress intensity factors for FRP-strengthened double-edged cracked steel plates can be calculated using the proposed expressions instead of by numerical methods. As a result, the crack growth behavior and crack growth life of FRP-strengthened double-edged cracked steel plates can be predicted conveniently. Moreover, the proposed expressions can also be used to develop the expressions of the stress intensity factor for FRP-strengthened steel beams with double-edged cracks in tension flange. 

## 2. Geometry of Specimens

**Figure 1:**
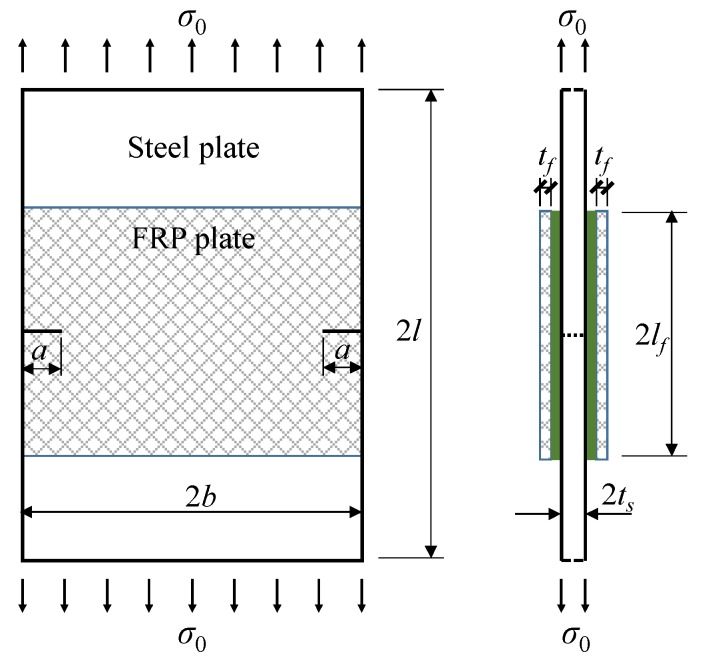
Geometric schematic of the specimen.

A geometric schematic of the specimens is presented in Figure 1. The length and width of the steel plate were 2*l* and 2*b*, respectively. The thickness of the steel plate was 2*t_s_*. Two through-thickness edged cracks were created on two sides of the steel plate. The length of each edged crack was *a*. Although the crack in this study was simplified to an ideal configuration without considering randomness in the crack geometry, such a simplification is typically used in the field of the fracture mechanics [2] where stress intensity factor solutions have only been developed for some ideal crack configurations. Moreover, a previous study [28] conducted by the current authors showed that the crack growth of FRP-strengthened double-edged cracked steel beam could be approximately predicted by assuming that the cracks at two edges are identical, indicating that such a simplification has practical significance. The FRP plates were bonded on the two sides of the steel plate. The FRP bond length was 2*l_f_,* and the bond width was the same as the width of the steel plate, i.e., 2*b*. The thickness of the FRP on each side was *t_f_*. A uniform adhesive layer was assumed, and the thickness was *t_a_*.

## 3. Theoretical Study on the Stress Intensity Factor

### 3.1. Stress Intensity Factor for the Cracked Plates Without Strengthening

According to linear elastic fracture mechanics, the stress intensity factor at the crack tip of an infinite center-cracked plate under tension for mode I crack opening is expressed by

(1) $$K=\sigma_{0}\sqrt{\pi a},$$

where *K* is the stress intensity factor for mode I crack opening; *σ*_0_ is the remote-field tension stress applied on the steel plate; and *a* is one-half of the central crack length.

For some finite cracked plates, the stress intensity factor can be calculated according to stress intensity factor handbooks [27]. The unified expression is given by

(2) $$K=F\cdot \sigma_{0}\sqrt{\pi a},$$

where *F* is the general correction factor that considers the effects of stress gradient, crack shape, surface crack, finite thickness and width of plate, and the eccentricity of crack against the central axis of the plate, which is given by [39]

(3) $$F=F_{g}\cdot F_{e}\cdot F_{s}\cdot F_{t}\cdot F_{h},$$

where *F_g_* is the correction factor for the stress gradient; *F_e_* is the correction factor for the crack shape; *F_s_* is the correction factor for a surface crack; *F_t_* is the correction factor for the finite thickness and width; and *F_h_* is the correction factor for the eccentricity of a crack against the central axis of the plate. For example, for finite-size plates with a through-thickness center-crack under uniform tension stress, *F_g_* = *F_e_* = *F_s_* = *F_h_* = 1. It can be seen that the values of the stress intensity factor for the finite cracked plates can be obtained by multiplying by the correction factor *F*. It follows that the stress intensity factor solution for the infinite center-cracked plate is the basis of the solutions for other finite cracked plates. Based on this concept, the stress intensity factor solution for the infinite center-cracked steel plate strengthened with FRP plates may serve as the fundamental part of the solution for FRP-strengthened cracked steel plates. Hence, the stress intensity factor solution for the infinite center-cracked steel plate strengthened with FRP plates is presented first.

### 3.2. Stress Intensity Factor for the Infinite Center-Cracked Steel Plate Strengthened with FRP Plates

For an infinite FRP-strengthened center-cracked steel plate subjected to remote-field tension stress, *σ*_0_, the remote-field tension stress, *σ*_0_, can be equivalent to the uniform tension stress, *σ*_s_, acting on the two surfaces of the center-crack through the superposition principle [34,35]. The uniform tension stress, *σ*_s_, is equal to the normal stress along the prospective crack path in the strengthening zones of the FRP-strengthened uncracked steel plate [34,35]. In order to calculate the stress intensity factors, the uniform tension stress, *σ_s_*, should be first obtained. 

**Figure 2:**
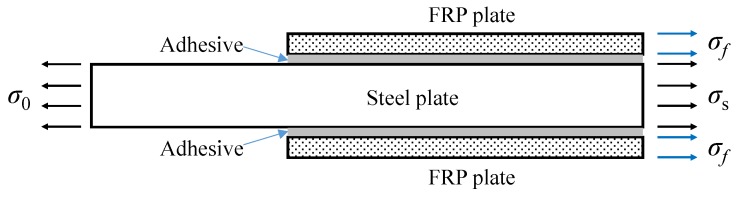
Stress components of the infinite uncracked steel plate strengthened with fiber reinforced polymer (FRP) plates.

For an infinite FRP-strengthened uncracked steel plate under the remote-field uniform tension stress, *σ*_0_, the external force is shared by the steel plate, adhesive layer, and FRP plates. In order to simplify the derivation of tension stress, *σ*_s_, two assumptions were employed: (1) the tension stress shared by the adhesive layer was very small and was neglected; and (2) there were no force-lag effects between the FRP and steel plate, which demonstrated that the tension strains of the FRP and steel plate were identical. Figure 2 presents the stress components of the FRP-strengthened uncracked steel plate. Based on the force equilibrium, the following equation can be easily obtained:

(4) $$\sigma_{0}A_{s}=\sigma_{s}A_{s}+2\sigma_{f}A_{f},$$

where *σ*_0_ is the remote-field uniform tension stress applied on the steel plate; *A_s_* is the cross-sectional area of the steel plate; *σ_s_* is the tension stress along the prospective crack path in the strengthened zone of the steel plate; *σ_f_* is the tension stress of the FRP plates; and *A_f_* is the cross-sectional area of FRP plates bonded on each side of the steel plate. The tension stress, *σ_s_*, in the strengthening zone of the steel plate can be derived with

(5) $$\sigma_{s}=\frac{\sigma_{0}}{1+S},$$

where *S* is the stiffness ratio between the FRP plate and the steel plate, which can be approximately expressed by

(6) $$S=\frac{2E_{f}A_{f}}{E_{s}A_{s}}=\frac{E_{f}t_{f}}{E_{s}t_{s}},$$

where *E_s_* and *E_f_* are the Young’s moduli of the steel and the FRP plate, respectively.

It can be seen that the tension stress in the strengthening zones can be reduced by bonding the FRP plates, and the reduction mainly depends on the stiffness ratio, *S*. 

**Figure 3:**
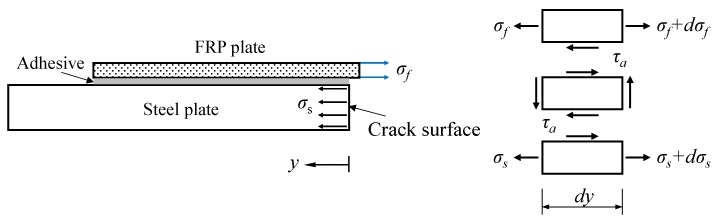
Stress analysis of the infinite center-cracked steel plate strengthened with FRP plates.

For the infinite center-cracked steel plate strengthened with FRP plates, both the geometry size and crack length are infinite. As a consequence, the stress state approximately conforms to the plane strain condition; thus, the 3D problem was simplified to the plane problem. Considering the symmetrical conditions, the one-quarter model is shown in Figure 3. Two assumptions were employed to simplify the analysis: (1) the adhesive layer was only subjected to shear deformation, and the shear stress was uniformly distributed along the adhesive thickness; (2) the steel plate and FRP plate were the elastic continuum, and the flexural deformation of the steel plate and FRP plate was neglected.

The equilibrium equations for the FRP plate and the steel plate can be expressed, respectively, by

(7) $$\frac{d\sigma_{f}}{dy}-\frac{\tau_{a}}{t_{f}}=0,$$

(8) $$\frac{d\sigma_{s}}{dy}+\frac{\tau_{a}}{t_{s}}=0,$$

where *τ_a_* is the shear stress in the adhesive layer. Under the plane strain state, the stress–displacement relationships for the FRP plate and the steel plate respectively are

(9) $$\sigma_{f}=\frac{E_{f}}{1-\upsilon_{f}^{2}}\varepsilon_{f}=\frac{E_{f}}{1-\upsilon_{f}^{2}}\frac{du_{f}}{dy},$$

(10) $$\sigma_{s}=\frac{E_{s}}{1-\upsilon_{s}^{2}}\varepsilon_{s}=\frac{E_{f}}{1-\upsilon_{s}^{2}}\frac{du_{s}}{dy},$$

where *ν_f_* is the Poisson’s ratio of the FRP plate; *u_f_* is the longitudinal displacement of the FRP plate along the *y* axis; *ν_s_* the Poisson’s ratio of the steel plate; and *u_s_* the longitudinal displacement of the steel plate along the *y* axis. 

Substituting Equations (9) and (10) into Equations (7) and (8) can lead to

(11) $$\frac{d^{2}u_{f}}{dy^{2}}-\frac{d^{2}u_{s}}{dy^{2}}=\tau_{a}\left(\frac{1-\upsilon_{f}^{2}}{E_{f}t_{f}}+\frac{1-\upsilon_{s}^{2}}{E_{s}t_{s}}\right).$$

According to the deformation compatibility between the FRP plate, adhesive layer, and steel plate, the following equation can be obtained:

(12) $$\gamma_{a}=\frac{u_{f}-u_{s}}{t_{a}},$$

where *γ_a_* is the shear strain of the adhesive layer. The relationship between the shear stress and shear strain can be expressed by

(13) $$\tau_{a}=G_{a}\gamma_{a}=G_{a}\frac{u_{f}-u_{s}}{t_{a}},$$

where *G*_a_ is the shear modulus of the adhesive. Combining Equations (11) and (13), the governing differential equation for the shear stress in the adhesive layer can be derived by

(14) $$\frac{d^{2}\tau_{a}}{dy^{2}}-\lambda^{2}\tau_{a}=0,$$

where

(15) $$\lambda^{2}=\frac{G_{a}}{t_{a}}\left(\frac{1-\upsilon_{f}^{2}}{E_{f}t_{f}}+\frac{1-\upsilon_{s}^{2}}{E_{s}t_{s}}\right).$$

By solving the above differential equation, the expression of the shear stress was obtained [35]:

(16) $$\tau_{a}=\tau_{a}^{\max}e^{-\lambda y},$$

where *τ_a_*^max^ is the maximum shear stress at the end of the adhesive layer.

Based on the shear stress distribution along the *y* axis in the adhesive layer, the upper bound of the stress intensity factor for the infinite center-cracked steel plate strengthened with double-sided FRP plates was derived by Rose and Wang [35]:

(17) $$K=\frac{\sigma_{s}}{\sqrt{k}},$$

where *σ_s_* is calculated according to Equation (5), and the parameter *k* is expressed by

(18) $$k=\frac{S\lambda }{\left(1+S)(1-\upsilon_{s}^{2}\right)}.$$

In order to formulate a similar form to the classical expression of the stress intensity factor, as shown in Equation (1), Equation (17) can be rewritten as:

(19) $$K=\sigma_{s}\sqrt{\pi c},$$

where

(20) $$c=\frac{1}{\pi k}=\frac{\left(1+S\right)}{S}\cdot \frac{1-\upsilon_{s}^{2}}{\pi \lambda }.$$

Equation (19) was derived based on the infinite crack length, so the obtained stress intensity factor is the upper bound for the infinite center-cracked steel plate strengthened with double-sided FRP plates [35]. When the crack is very short, the stress intensity factor can be approximately calculated by Equation (1). Such approximate calculations are conservative due to the strengthening effect of the FRP plates; thus, the calculation results can be considered as the lower bound of the stress intensity factor for the infinite center-cracked steel plate strengthened with double-sided FRP plates [34]. For an arbitrary length, *a*, the following expression was formulated to calculate the stress intensity factor for the infinite center-cracked steel plate strengthened with double-sided FRP plates [34]:

(21) $$K=\sigma_{s}\sqrt{\frac{\pi ac}{a+c}}=\sqrt{\frac{c}{a+c}}\cdot \sigma_{s}\sqrt{\pi a}.$$

It can be seen that the calculated results from Equation (21) approach the results from Equation (1) when crack length *a* is far less than *c*. When the crack length is very long, the calculated results from Equation (21) approach the results from Equation (19). This demonstrates that Equation (21) conforms to the upper and lower bounds. 

By substituting Equation (5) into Equation (21), the stress intensity factor at the crack length of *a* under remote-field tension stress, *σ*_0_, for the infinite center-cracked steel plate strengthened with double-sided FRP plates can be calculated with the following expression:

(22) $$K=\frac{1}{1+S}\cdot \sqrt{\frac{c}{a+c}}\cdot \sigma_{0}\sqrt{\pi a}=\alpha_{1}\cdot \alpha_{2}\cdot \sigma_{0}\sqrt{\pi a},$$

where

(23) $$\alpha_{1}=\frac{1}{1+S},$$

(24) $$\alpha_{2}=\sqrt{\frac{c}{a+c}}.$$

Compared with the stress intensity factor solution for the infinite center-cracked steel plate, two correction factors, i.e., *α*_1_ and *α*_2_, were introduced in the stress intensity factor solution for the FRP-strengthened infinite center-cracked steel plates to consider the FRP strengthening effect. The values of both correction factors were less than one, demonstrating that FRP strengthening could decrease the stress intensity factors. The strengthening effect of FRP plates mainly arises from the reduction of the stress and constraint to the crack opening [27,34]. The first correction factor, *α*_1_, represents the reduction effect of the remote-field tension stress, which mainly depends on the stiffness ratio, *S*. A larger stiffness ratio, *S*, can result in smaller stress intensity factor values. The second correction factor, *α*_2_, indicates the constraint effect to the crack, which mainly depends on the crack length, the stiffness ratio, *S*, the shear modulus, and the thickness of the adhesive. The stress intensity factor can be decreased by increasing the stiffness ratio, *S*, and the adhesive shear modulus, and/or reducing the adhesive thickness.

### 3.3. Stress Intensity Factor for the Double-Edged Cracked Steel Plate Strengthened with FRP Plates

Based on the concept presented in Section 3.1, the stress intensity factor solution for the FRP-strengthened double-edged cracked steel plate was formulated by modifying Equation (22). Finally, the following expressions were formulated by introducing two correction factors to calculate the stress intensity factors for the double-edged cracked steel plate strengthened with FRP plates:

(25) $$K=\beta \left(a,b,S,...\right)\cdot f(a,b)\cdot \frac{1}{1+S}\cdot \sqrt{\frac{c}{a+c}}\cdot \sigma_{0}\sqrt{\pi a},$$

(26) $$f=\left[1-0.025{\left(\frac{a}{b}\right)}^{2}+0.06{\left(\frac{a}{b}\right)}^{4}\right]\sqrt{\sec\frac{\pi a}{2b}},$$

where *f* is the geometric correction factor of the unstrengthened cracked plate to allow consideration of the effect of the specimen’s width, and *β* is the additional correction factor which is used to further consider the effect of FRP reinforcements. It is noted that although coefficients *α*_1_ and *α*_2_ consider the effect of FRP reinforcements, coefficient *α*_2_ is only suitable for the FRP-strengthened infinite cracked plate. Therefore, for the finite cracked plate, the additional correction factor *β* was introduced to correct coefficient *α*_2_. Given the main influencing factors of coefficient *α*_2_, the additional correction factor, *β*, may depend on the crack length, specimen width, stiffness ratio, *S*, and adhesive properties. In order to determine the main influencing factors of additional correction factor *β*, parametric investigations should be conducted. Considering the complexity of the stress intensity factors at the crack tip, an exact theoretical method is an almost intractable task in parametric investigations. Methods based on numerical simulations for determining the correction factor have been widely used in previous studies on stress intensity factors [2,34,38,40,41]. Therefore, FE simulations were carried out and are described in the following section.

## 4. Finite Element Modeling

This section describes the FE analysis, which was conducted to investigate the stress intensity factors fordouble-edged cracked steel plates strengthened with FRP plates. The FE results were used to investigate the main influencing factors on the stress intensity factors and additional correction factor *β*, to calibrate additional correction factor *β*.

### 4.1. The Geometry and Material Parameters of the Specimens

A total of four specimens were selected for the numerical parametric investigations, as listed in Table 1. The height and thickness of the steel plate for all four specimens were 700 mm and 10 mm, respectively. The four specimens had different widths, i.e., 90 mm, 120 mm, 150 mm, and 200 mm, respectively. P-90 denotes the specimen with a width of 90 mm. Different crack lengths were set for each specimen to simulate the different degrees of damage before strengthening. The crack lengths changed at an interval of 5 mm for the four specimens. The effects of the FRP thickness, FRP Young’s modulus, the adhesive thickness, and the adhesive shear modulus on the stress intensity factors were analyzed. The variable values of these parameters covered a wide range, as summarized in Table 1. It should be noted that existing studies show that the FRP bond length has no effect on the stress intensity factor when the bond length is longer than the effective bond length [42,43]. Considering that an effective bond length is usually ensured in practical applications, the bond length was not considered as a variable in the FE modeling. The different combinations of FRP thickness and Young’s modulus provided different stiffness ratios, *S*. The Young’s modulus and the Poisson’s ratio of the steel were 206 GPa and 0.3, respectively, which remained unchanged in the parametric investigations for all specimens. The reference material properties of the FRP plate and the adhesive are listed as follows: the thickness and Young’s modulus of the FRP plate were 1.4 mm and 165 GPa, respectively; the Poisson’s ratio of the FRP plate was 0.28; the thickness and shear modulus of the adhesive layer were 1.0 mm and 900 MPa, respectively; and the Poisson’s ratio of the adhesive was 0.35. When investigating the effect of one variable, the values of the other variables remained unchanged during the parametric investigations. Two extra specimens with different geometric sizes, i.e., P-200* and P-300*, are also listed in Table 1. These were used to validate the proposed expressions of the stress intensity factors.

### 4.2. FE Models

**Figure 4:**
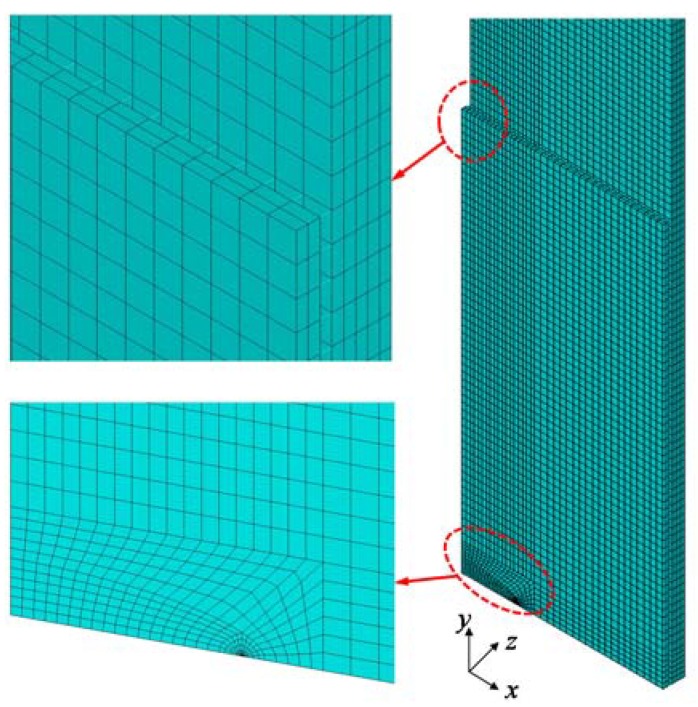
Typical finite element model.

The 3D linear elastic FE models were developed using ANSYS software to calculate the stress intensity factors. One-quarter of the specimens were modeled considering symmetry conditions and thus, symmetrical boundary conditions were applied to the nodes on symmetrical planes. A uniform stress of 150 MPa was applied to the FE models at the longitudinal end of the steel plate. The typical FE meshes are shown in Figure 4. In the FE models, the FRP plate and steel plate were modeled using the eight-node 3D solid element SOLID45, which has three translational degrees of freedom at each node. The mesh refinement was set in the vicinity of the crack tip. Twelve elements were meshed around the circumferential direction in the FE models, as shown in Figure 4. The element length in the vicinity of crack tip was set to 1/40 of the crack length based on the mesh convergence analysis. Using the assumption that no relative slip occurred the FRP–adhesive interface or the adhesive–steel interface, the adhesive layer was modeled using the linear spring element COMBIN14. To simulate the axial and shear deformation of the adhesive layer, three COMBIN14 elements were installed between each node pair corresponding to the FRP–adhesive interface and the adhesive–steel interface. The shear and axial spring constants were obtained by [31]

(27) $$K_{i}=\frac{G_{a}A_{a}}{t_{a}},$$

(28) $$K_{z}=\frac{2(1-\nu_{a})G_{a}A_{a}}{(1-2\nu_{a})t_{a}},$$

where *K_i_* (*i* = *x*, *y*) and *K_z_* are the shear and axial spring constants, respectively; and *A_a_* is the adhesive area represented by the responding spring. In the FE models, all of the materials were assumed to be elastic, and linear elastic analyses were conducted. The virtual crack closure method [44] was used to calculate the energy release rate of the crack tip. Using this method, quarter-point singular elements were not necessary in the models, and the calculated results were insensitive to the FE mesh sizes [27,44]. The energy release rate was obtained conveniently by picking out the elemental nodal forces at the crack tip and the nodal displacements behind the crack tip. The stress intensity factor was obtained according to the following equation:

(29) $$K=\sqrt{G_{I}\cdot E_{s}},$$

where *G*_I_ is the energy release rate in the fracture mode I; and *E_s_* is the Young’s modulus of the steel.

## 5. FE Results and Discussion

This section describes the investigation of the effects of the FRP thickness, FRP Young’s modulus, adhesive thickness, and adhesive shear modulus on the stress intensity factors based on numerical results. As similar stress intensity factor trends were observed for all four specimens, only the results of specimen P-150 are presented here for analysis.

### 5.1. Effect of the FRP Thickness

**Figure 5:**
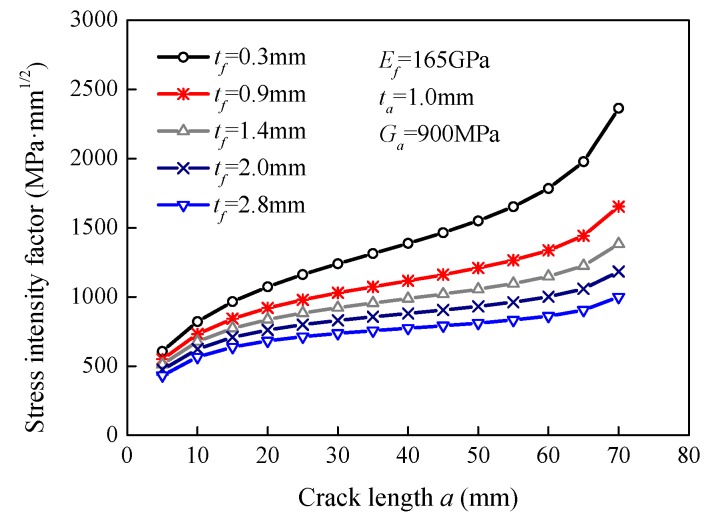
Effect of the FRP thickness on the stress intensity factor.

The steel plates strengthened with different FRP thicknesses, i.e., 0.3 mm, 0.9 mm, 1.4 mm, 2.0 mm, and 2.8 mm, were modeled to investigate the effect of FRP thickness on the stress intensity factor. Figure 5 presents the variation in stress intensity factor with the crack length at different FRP thicknesses. It is obvious that the stress intensity factor increased gradually with the increase in the crack length for each of the curves. At the same crack length, the stress intensity factor significantly reduced with an increase in the FRP thickness. When the crack length, *a*, was 20 mm, the stress intensity factor reduced from 1073.5 MPa·mm^1/2^ to 682.5 MPa·mm^1/2^, i.e., a reduction of 36.4%, as the FRP thickness increased from 0.3 mm to 2.8 mm. When the crack length, *a*, was 50 mm, the FRP plate with a thickness of 2.8 mm had a reduced stress intensity factor by 47.7% compared with the FRP plate with a thickness of 0.3 mm. Therefore, the strengthening effect was more effective with thicker FRP plates, because thicker FRP plates are able to share more stress.

### 5.2. Effect of the FRP Young’s Modulus

**Figure 6:**
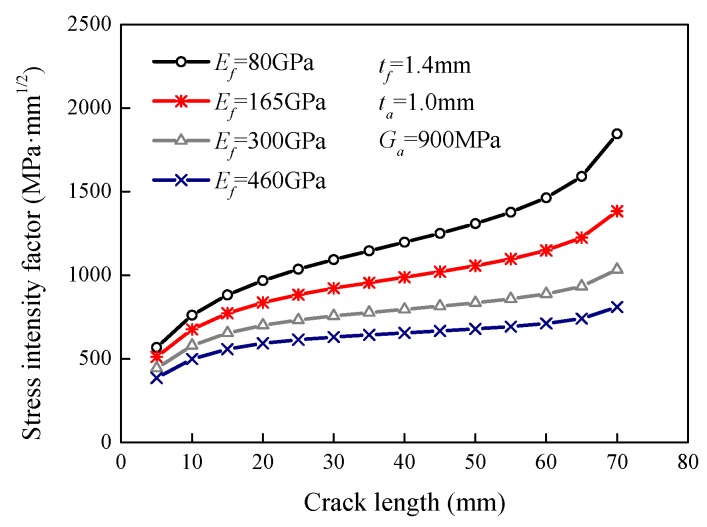
Effect of the FRP Young’s modulus on the stress intensity factor.

Figure 6 plots the variation curves of the stress intensity factor versus the crack length for specimens with different FRP plates. FRP plates with Young’s moduli of 80 GPa, 165 GPa, 300 GPa, and 460 GPa were used to strengthen the steel plates. The FE results indicated that the higher the FRP modulus was, the smaller the stress intensity factor was at the same crack length. For the 20 mm crack, the stress intensity factor decreased from 968.3 MPa·mm^1/2^ to 592.6 MPa·mm^1/2^, i.e., decreased by 38.8%, when the FRP modulus increased from 80 GPa to 460 GPa. For the 50 mm crack, the increase in the Young’s modulus from 80 GPa to 460 GPa reduced the stress intensity factor by 48.1%. It can be concluded that the FRP modulus has a considerable effect on the reduction in the stress intensity factor as a higher modulus can make FRP plates share more stress. The investigations presented in Section 5.1 and Section 5.2 show that an increase in the stiffness ratio, *S*, can reduce the stress intensity factor.

### 5.3. Effect of the Adhesive Thickness

**Figure 7:**
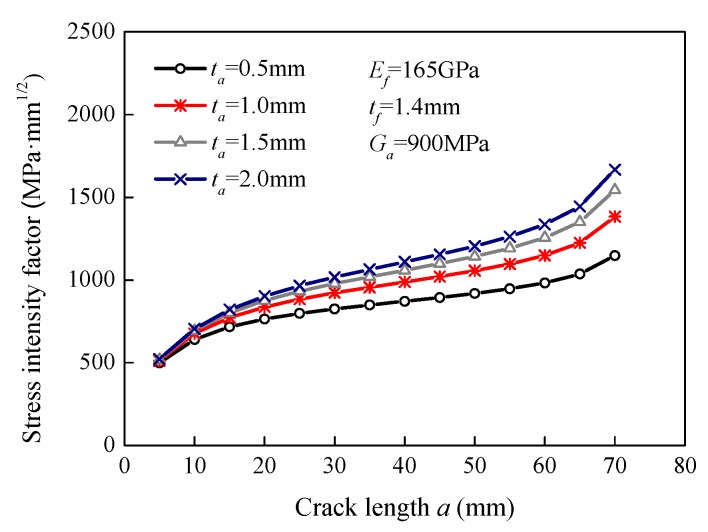
Effect of the adhesive thickness on the stress intensity factor.

Figure 7 illustrates the effect of the adhesive thickness on the stress intensity factor. The modeled adhesive thicknesses were 0.5 mm, 1.0 mm, 1.5 mm, and 2.0 mm. It can be seen that the stress intensity factor increased with an increase in the adhesive thickness. When the adhesive thickness increased from 0.5 mm to 2.0 mm, the stress intensity factor increased from 764.3 MPa·mm^1/2^ to 902.3 MPa·mm^1/2^ at the 20 mm long crack, i.e., increased by 18.1%. The stress intensity factor increased by 31.2% at the 50 mm long crack. It can be seen from Equations (27) and (28) that an increase in the adhesive thickness can reduce the axial and shear stiffness of the spring elements, resulting in the reduction in the efficacy of stress transfer between the steel and FRP plates. Therefore, a thinner adhesive layer could result in a smaller stress intensity factor. However, the thinner adhesive could lead to a lower bond strength and thus, could cause FRP debonding based on previous studies on interfacial bond behavior [45].

### 5.4. Effect of the Adhesive Shear Modulus

**Figure 8:**
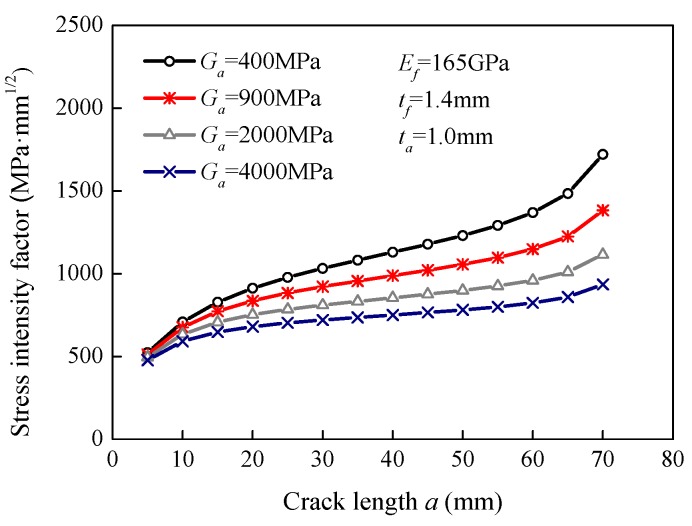
Effect of the adhesive shear modulus on the stress intensity factor.

Specimens with different values of adhesive shear modulus were modeled to investigate the effect of the adhesive shear modulus on the stress intensity factor. The values of the shear modulus in the FE models were 400 MPa, 900 MPa, 2000 MPa, and 4000 MPa. Figure 8 shows the variation in the stress intensity factor in terms of the crack length at different values of shear modulus. The FE results show that the stress intensity factor could be reduced by a higher adhesive shear modulus. When the crack length was 20 mm, the stress intensity factors decreased from 912.4 MPa·mm^1/2^ to 680.5 MPa·mm^1/2^, i.e., decreased by 25.4% when the shear modulus increased from 400 MPa to 4000 MPa. When the crack length was 50 mm, the increase in the shear modulus from 400 MPa to 4000 MPa reduced the stress intensity factors by 36.5%. Moreover, it should be noted that a high adhesive shear modulus can also cause the risk of FRP debonding failure [45].

## 6. Development of Correction Factor *β*

Based on the FE results for the stress intensity factor, the values of additional correction factor *β* were obtained by rewriting Equation (25):

(30) $$\beta \left(a,b,S,...\right)=\frac{K_{FE}}{f\cdot \frac{1}{1+S}\cdot \sqrt{\frac{c}{a+c}}\cdot \sigma_{0}\sqrt{\pi a}},$$

where *K_FE_* is the value of the stress intensity factors calculated by the FE method. The effects of the specimen width, crack length, stiffness ratio, adhesive thickness, and adhesive shear modulus on additional correction factor *β* are discussed in the following sections.

**Figure 9:**
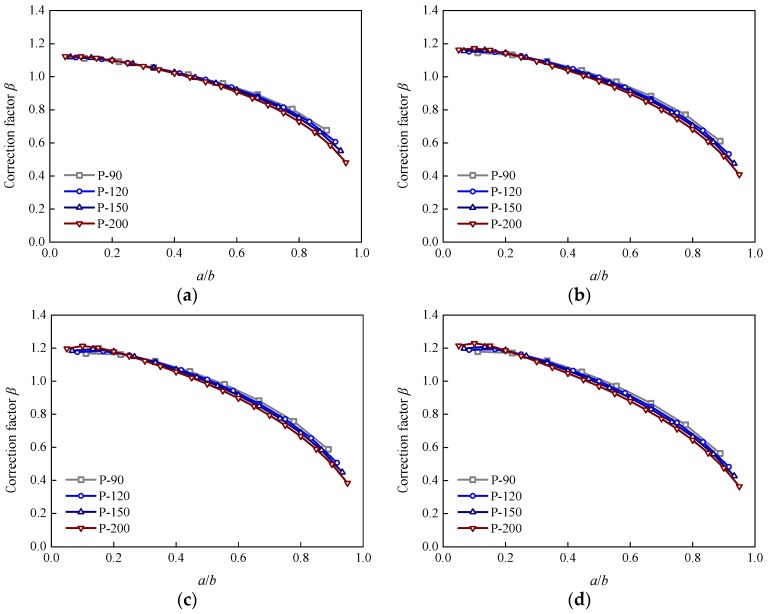
Variation inf correction factor *β* with *a*/*b:* (**a**) *S* = 0.05; (**b**) *S* = 0.22; (**c**) *S* = 0.45; (**d**) *S* = 0.63.

**Figure 10:**
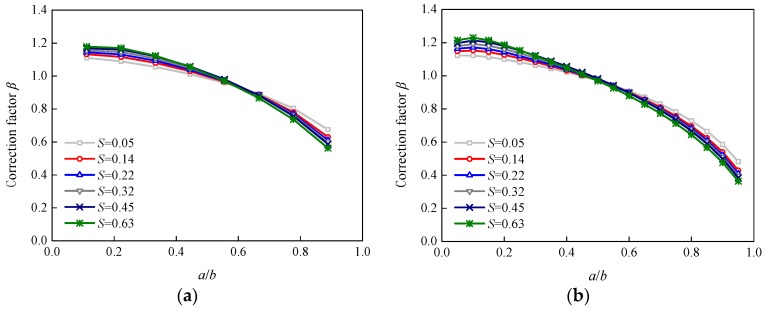
Variation in correction factor *β* with the stiffness ratio, *S*: (**a**) Specimen P-90; (**b**) Specimen P-200.

To evaluate the effects of specimen width and crack length for different specimens together, a variable was defined as the ratio between the crack length, *a*, and half-width, *b*, of the specimen, i.e., *a*/*b*. Figure 9 plots the variation in additional correction factor *β* with the *a*/*b* at different stiffness ratios, *S*. It can be seen that the variation trend was similar at different stiffness ratios, *S*. The values of additional correction factor *β* reduced gradually with an increase in *a*/*b*. The reduction became more and more significant with an increase in *a*/*b*. Moreover, a comparison of the results for different specimens showed that the specimen width had little effect on additional correction factor *β* when the values of *a*/*b* were the same. The values of additional correction factor *β* were smaller for the wider specimens at the same *a*/*b*; however, the difference was very small. Figure 10 presents the effect of the stiffness ratio, *S*, on additional correction factor *β* for specimens P-90 and P-200. The trends for the effect of the stiffness ratio, *S*, were similar for the other two specimens. It was found that the effect of the stiffness ratio, *S*, on additional correction factor *β* were similar for the different specimens. When the values of *a*/*b* were smaller, the values of additional correction factor *β* increased gradually with an increase in the stiffness ratio, *S*. However, the values of additional correction factor *β* reduced with an increasing stiffness ratio, *S*, when the values of *a*/*b* became larger. Generally, changes in additional correction factor *β* with the stiffness ratio, *S*, were not very obvious when compared with the effect of *a*/*b*.

**Figure 11:**
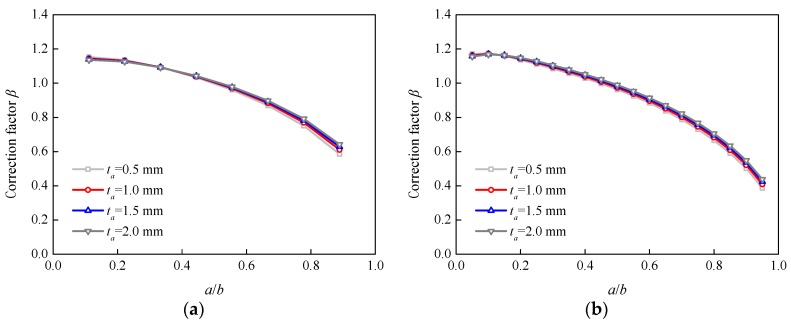
Variations in correction factor *β* with the adhesive thickness: (**a**) Specimen P-90; (**b**) Specimen P-200.

**Figure 12:**
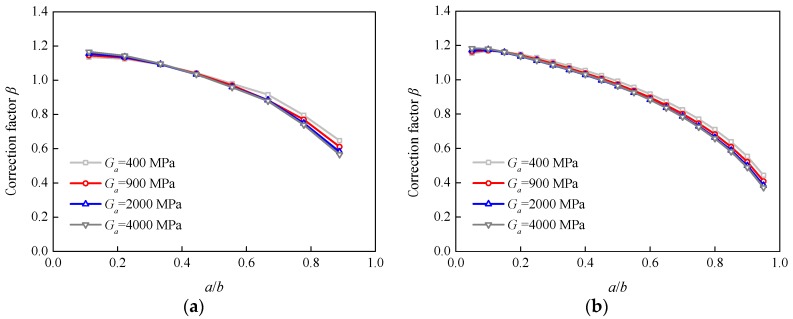
Variation in correction factor *β* with the adhesive shear modulus: (**a**) Specimen P-90; (**b**) Specimen P-200.

Figure 11 and Figure 12 show the effects of the adhesive thickness and shear modulus on additional correction factor *β* for specimens P-90 and P-200, respectively. The effect trends of the adhesive thickness and shear modulus were similar for the other two specimens. It can be seen that the adhesive thickness and shear modulus had very little effect on the values of additional correction factor *β*.

Based on the above investigation, to simplify the expression of additional correction factor *β*, the effects of the adhesive thickness and shear modulus were neglected, and only the effects of *a*/*b* and the stiffness ratio, *S*, were considered in the proposed expression. Finally, the expression of additional correction factor *β* was formulated as

(31) $$\beta =1+\left[\eta_{1}+\eta_{2}\left(\frac{a}{b}\right)+\eta_{3}{\left(\frac{a}{b}\right)}^{2}\right]S^{\eta_{4}}\;$$

where *η*_1_, *η*_2_, *η*_3_, and *η*_4_ are the coefficients to be determined. Based on the FE data, the best-fit values of the four coefficients were 0.187, 0.13, −1.04, and 0.12, respectively. By substituting the four values into Equation (31), the expression of additional correction factor *β* for double-edged cracked steel plates strengthened with double-sided FRP plates was obtained with

(32) $$\beta =1+\left[0.187+0.13\frac{a}{b}-1.04{\left(\frac{a}{b}\right)}^{2}\right]S^{0.12}.$$

Therefore, according to Equations (25), (26), and (32), the stress intensity factor for double-edged cracked steel plates strengthened with double-sided FRP plates can be calculated. It was found that the proposed expression of the stress intensity factor is a function of the applied stress, crack length, the ratio between the crack length and the width of the steel plate, *a*/*b*, the stiffness ratio, *S*, the adhesive shear modulus, and the adhesive thickness.

## 7. Verification of the Proposed Equations

**Figure 13:**
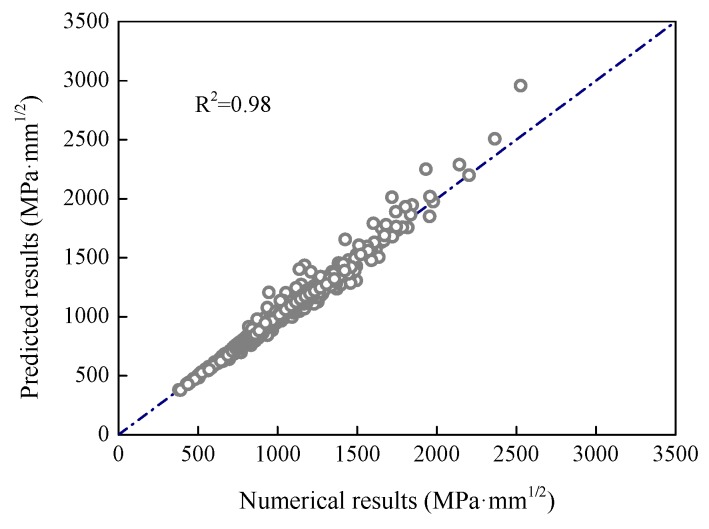
Comparison of the theoretical and numerical results.

According to the proposed equations, the stress intensity factors of the specimens were calculated. Figure 13 presents the comparisons of the theoretical and numerical results for the four specimens. It was found that the theoretical results had good agreement with the numerical results for all four specimens. The mean of the ratios between the theoretical and numerical results for all specimens was 1.0, and the coefficient of variation (COV) was 0.04. 

**Figure 14:**
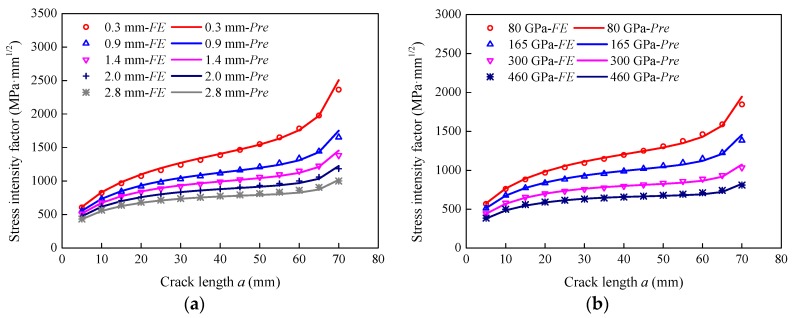

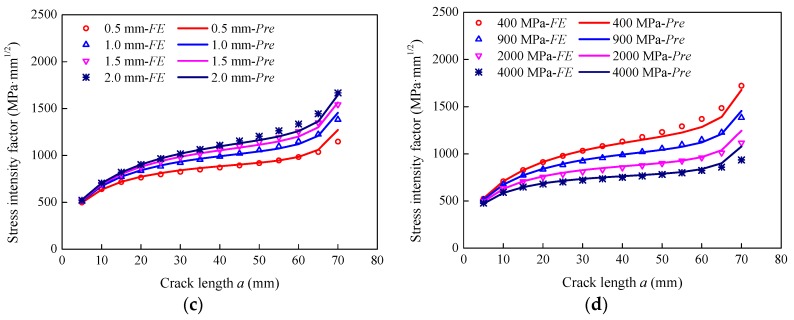
Comparison of the theoretical and numerical results for specimen P-150: (**a**) effect of the FRP thickness; (**b**) effect of the FRP modulus; (**c**) effect of the adhesive thickness; and (**d**) effect of the adhesive shear modulus.

**Figure 15:**
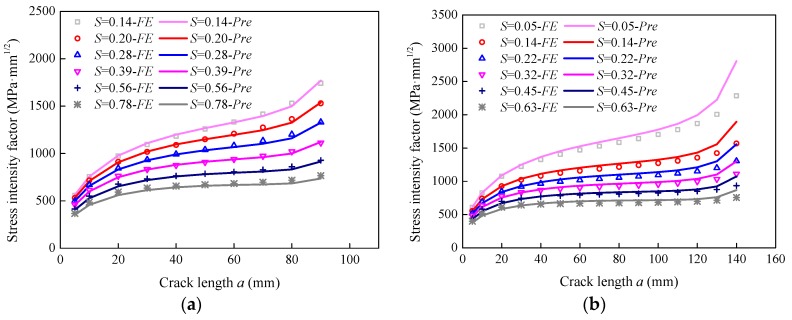
Comparison of the theoretical and numerical results: (**a**) Specimen P-200* and (**b**) Specimen P-300*.

Figure 14 shows the comparison between the theoretical and numerical results for specimen P-150. It can be seen that the theoretical results had good agreement with the numerical results at different values of FRP thickness, FRP modulus, adhesive thickness, and adhesive modulus. In order to further validate the proposed equations, two extra specimens, i.e., P-200* and P-300*, were also modeled. The variables for both specimens are listed in Table 1. Figure 15 plots the comparison of the theoretical and numerical results for both specimens. It can be seen that the theoretical results also agreed well with the numerical results for both specimens. The above comparisons demonstrate that the proposed equations can predict the stress intensity factor for double-edged cracked steel plates strengthened with double-sided bonded FRP plates with reasonable accuracy.

## 8. Conclusions

Theoretical and numerical studies were carried out to investigate the stress intensity factor for the double-edged cracked steel plates strengthened with double-sided bonded FRP plates. Expressions of the stress intensity factor were proposed based on those for infinite center-cracked steel plates strengthened with double-sided bonded FRP plates. 3D FE modeling was conducted to investigate the main influencing factors on the stress intensity factor and the additional correction factor, *β*. The following conclusions were drawn:

- The stress intensity factor for the double-edged cracked steel plates strengthened with FRP plates decreased with an increase in the FRP thickness, the FRP Young’s modulus, and the adhesive shear modulus. An increase in the adhesive thickness could increase the stress intensity factor.
- The additional correction factor, *β*, was mainly affected by the ratio between the crack length and the specimen width and the stiffness ratio. The adhesive thickness and shear modulus had little effect on additional correction factor *β*. Additional correction factor *β* was formulated and calibrated based on the FE results.
- The stress intensity factor for the double-edged cracked steel plates strengthened with FRP plates can be calculated based on the proposed Equations (25), (26), and (32). The proposed expressions of the stress intensity factor were a function of the applied stress, crack length, the ratio between the crack length and the width of the steel plate, *a*/*b*, the stiffness ratio, *S*, the adhesive shear modulus, and the adhesive thickness. The proposed expressions were shown to calculate stress intensity factors with reasonable accuracy.

## Figures and Tables

**Table 1:** The analyzed variables of the specimens.

Specimen	Steel Plate				FRP Plate		Adhesive	
	Length, 2*l* (mm)	Width, 2*b* (mm)	Thickness, 2*t_s_* (mm)	Crack Length, *a* (mm)	Thickness, *t_f_* (mm)	Young’s Modulus, *E_f_* (GPa)	Thickness, *t_a_* (mm)	Shear Modulus, *G_a_* (MPa)
P-90	700	90	10	5, 10, 15, 20, 25, 30, 35, 40	0.3, 0.9, 1.4, 2.0, 2.8	80, 165, 300, 460	0.5, 1.0, 1.5, 2.0	400, 900, 2000, 4000
P-120	700	120	10	5, 10, 15, 20, 25, 30, 35, 40, 45, 50, 55	0.3, 0.9, 1.4, 2.0, 2.8	80, 165, 300, 460	0.5, 1.0, 1.5, 2.0	400, 900, 2000, 4000
P-150	700	150	10	5, 10, 15, 20, 25, 30, 35, 40, 45, 50, 55, 60, 65, 70	0.3, 0.9, 1.4, 2.0, 2.8	80, 165, 300, 460	0.5, 1.0, 1.5, 2.0	400, 900, 2000, 4000
P-200	700	200	10	5, 10, 15, 20, 25, 30, 35, 40, 45, 50, 55, 60, 65, 70, 75, 80, 85, 90	0.3, 0.9, 1.4, 2.0, 2.8	80, 165, 300, 460	0.5, 1.0, 1.5, 2.0	400, 900, 2000, 4000
P-200 *	1000	200	16	5, 10, 20, 30, 40, 50, 60, 70, 80, 90,	1.4, 2.0, 2.8	165, 460	1.0	900
P-300 *	1000	300	10	5, 10, 20, 30, 40, 50, 60, 70, 80, 90, 100, 110, 120, 130, 140	0.3, 0.9, 1.4, 2.0, 2.8	165, 460	1.0	900

* For verification.
